# Climate-Driven Reshuffling of Species and Genes: Potential Conservation Roles for Species Translocations and Recombinant Hybrid Genotypes

**DOI:** 10.3390/insects5010001

**Published:** 2013-12-24

**Authors:** Jon Mark Scriber

**Affiliations:** 1 Department of Entomology, Michigan State University, East Lansing, Michigan, MI 48824, USA; E-Mail: scriber@msu.edu; Tel.:+1-517-432-1975; 2 McGuire Center for Lepidoptera and Biodiversity, Florida Museum of Natural History, University of Florida, Gainesville, FL 32611, USA

**Keywords:** climate change, biodiversity, translocation conservation, hybrid conservation, genetic introgression, hybrid speciation, hybrid extinction

## Abstract

Comprising 50%–75% of the world’s fauna, insects are a prominent part of biodiversity in communities and ecosystems globally. Biodiversity across all levels of biological classifications is fundamentally based on genetic diversity. However, the integration of genomics and phylogenetics into conservation management may not be as rapid as climate change. The genetics of hybrid introgression as a source of novel variation for ecological divergence and evolutionary speciation (and resilience) may generate adaptive potential and diversity fast enough to respond to locally-altered environmental conditions. Major plant and herbivore hybrid zones with associated communities deserve conservation consideration. This review addresses functional genetics across multi-trophic-level interactions including “invasive species” in various ecosystems as they may become disrupted in different ways by rapid climate change. “Invasive genes” (into new species and populations) need to be recognized for their positive creative potential and addressed in conservation programs. “Genetic rescue” via hybrid translocations may provide needed adaptive flexibility for rapid adaptation to environmental change. While concerns persist for some conservationists, this review emphasizes the positive aspects of hybrids and hybridization. Specific implications of natural genetic introgression are addressed with a few examples from butterflies, including transgressive phenotypes and climate-driven homoploid recombinant hybrid speciation. Some specific examples illustrate these points using the swallowtail butterflies (Papilionidae) with their long-term historical data base (phylogeographical diversity changes) and recent (3-decade) climate-driven temporal and genetic divergence in recombinant homoploid hybrids and relatively recent hybrid speciation of *Papilio appalachiensis* in North America. Climate-induced “reshuffling” (recombinations) of species composition, genotypes, and genomes may become increasingly ecologically and evolutionarily predictable, but future conservation management programs are more likely to remain constrained by human behavior than by lack of academic knowledge.

## 1. Introduction

**Figure 1:**
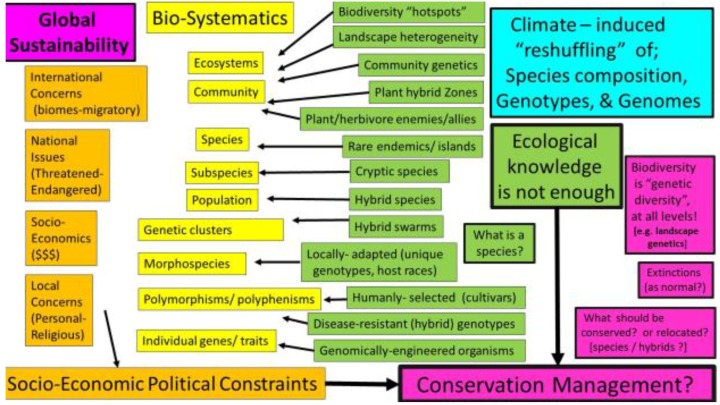
Some structural and functional aspects of insect conservation and diversity. Unsettled academic issues about bio-systematics persist (e.g., what is a species?), yet we know a tremendous amount about biodiversity genomes and genotypes to interspecific hybrid zones and community-level trophic interactions that determine distribution limits, abundance, and latitudinal diversity across global ecosystems. This review addresses some biological issues and impacts of climate-induced “reshuffling” (recombination) of species composition, genotypes, and genomes. However, future conservation management programs will not be constrained by our capacity for ecological/evolutionary predictions, but rather by our own human behavior. Ecologists and conservation managers need to work more closely with sociologists, economists, politicians and religious groups if we want a more sustainable world.

Today, conservation is about managing biodiversity at many levels (genomics of individuals, population genetics, community interactions and ecosystem/landscape genetics) in the face of rapid environmental change, rather than always trying to stabilize things into the future as they have been historically. Given the rapid rate of recent climate change [1], the future of conservation strategies may center upon enhancement of genetic diversity via community level hybridization (e.g., translocations of local endemics) and recognition, protection, and use of intra-specific and inter-specific hybridization (genetic introgression) to maintain and increase genetic variance within populations. This review will briefly address climate-induced changes in biodiversity patterns and compositional reshuffling at different structural levels of organization from ecosystems to genomes ([2,3,4,5]; Figure 1). Changing biotic interactions of species and differential competition and dispersal abilities in communities may affect the realization of range shift potentials as much as the abiotic changes, and these too need to be included with modelling of climate change impacts [6,7,8,9,10,11,12]. While many of the ecological responses of plants and animals to climatic change have been increasingly recognized, the associated genetically-based adaptive mechanisms remain largely understudied [13,14,15].

The examples used here will mostly deal with ectothermic insects and their abiotic and biotic environment, which is likely to be significantly altered by continuing rapid climate change during both the summer and winter seasons. It is important to realize that locally-adapted genotypes and other intraspecific “cryptic biodiversity” may be as important for conservation as endemic species [16]. For example, the use of morpho-species greatly underestimates the true genetic biodiversity losses due to climate change [17]. Intraspecific patterns of genetic diversity need to become a fundamental part of studies in biodiversity and biodiversity losses [16]. Reasons for addressing functional genetics across all structural classification levels from genotypes to ecosystems for conservation ecology and management are discussed below (Figure 1). The swallowtail butterflies (family Papilionidae) have provided an nexception group for studying responses to long term and short term climate changes across a broad geographic area, with multiple trait analyses, and one of the very first cases of animal hybrid speciation (see below).

**Figure 2:**
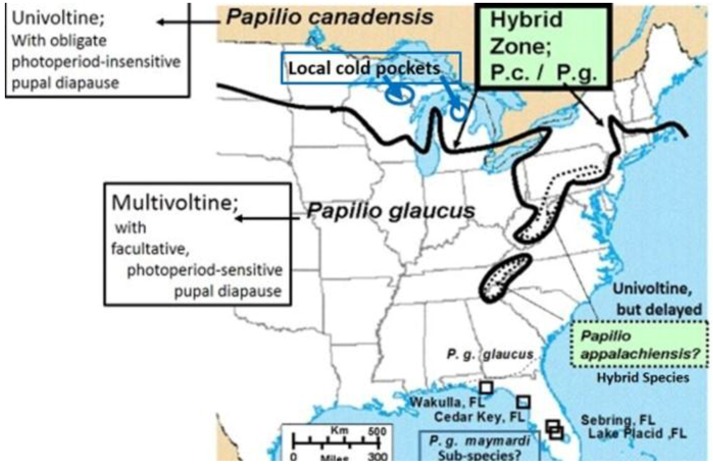
The historical tension zone of hybrid interactions across the North American Great Lakes region and down the Appalachian Mountains [326,329]. The dotted line delineates the geographic locations of the type specimens for the hybrid species, *P*. *appalachiensis* [26,99]. A purported subspecies (*P*. *g*. *maynardi*) occurs south of Florida’s northern border, with some unique adaptations [201,345,487].

**Figure 3:**
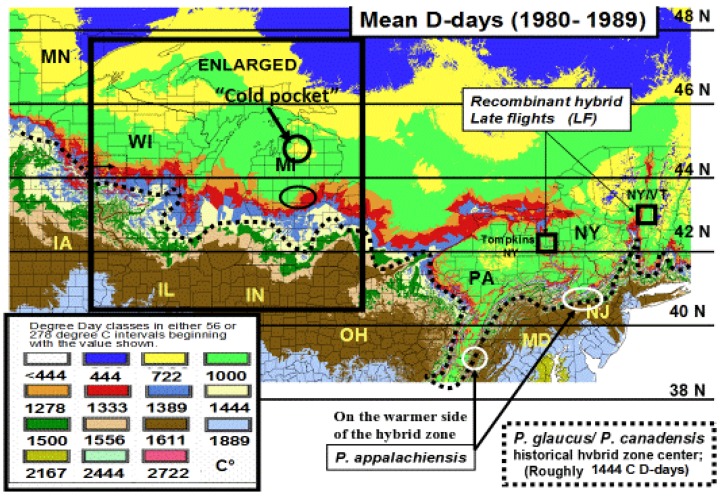
The thermal landscape of eastern North America showing the center of the historical hybrid zone (dotted line) between *Papilio glaucus* and *P*. *canadensis* as a function of the mean annual Degree-day accumulations above a base of 50 °F (=10 °C) for the decade from 1980–1989 (1960–1979 was very similar). The center of this zone (and northern limits of bivoltine potential in *P*. *glaucus*) is at approximately 2600 D-days F (or 1444 C D-days). The *P*. *appalachiensis* populations are shown on the warm side of the hybrid zone (in the mountains of Pennsylvania and West Virginia), while the recombinant “late-flight” hybrid populations have been described from central and eastern New York State, on the cool side of this thermally-defined hybrid zone. The Otsego county “climatic cold pocket” in northern Michigan is indicated here, and also in the enlargement (Figure 4).

In general, natural “adaptive introgression” in animals has been receiving increased attention in historical adaptive radiations [18]. Adaptive introgression also has potential use under new environmental challenges as well as in species “genetic rescue” of impoverished populations under past inbreeding or genetic drift [19,20,21,22,23]. Natural hybridization with adaptive radiation, with evolutionary divergence, incipient speciation, and hybrid speciation across the 1,500 km length of a major North American ecotone from the Great Lakes to New England will also be highlighted here (Figure 2). Rapid climate warming suggests that multiple parapatric origins of incipient (hybrid) species may occur on the cool side (Figure 3) of the historical (and thermally-defined) hybrid zone, while multiple and extensive extinctions of certain recombinant hybrids, and hybrid species may occur on the warm side of the hybrid zone, especially in Appalachian high mountain refuges [24]. A dynamic balance in creation of new genotypes (and incipient hybrid species) and their extinctions appears to be driven or mediated by recent climate changes, especially during the past 15 years [24], but possibly longer [25,26,27]. Among all swallowtail butterflies (>550 species), the number of species of conservation concern are more than 70 on islands, more than 50 in tropical dry and wet forests, and more than 20 in highlands [28]. Perhaps hybrid species and recombinant hybrid genotypes should also be considered. However, whether hybrids should be eligible for legal protection remains a heavily debated and heated issue, even today [29]. 

**Figure 4:**
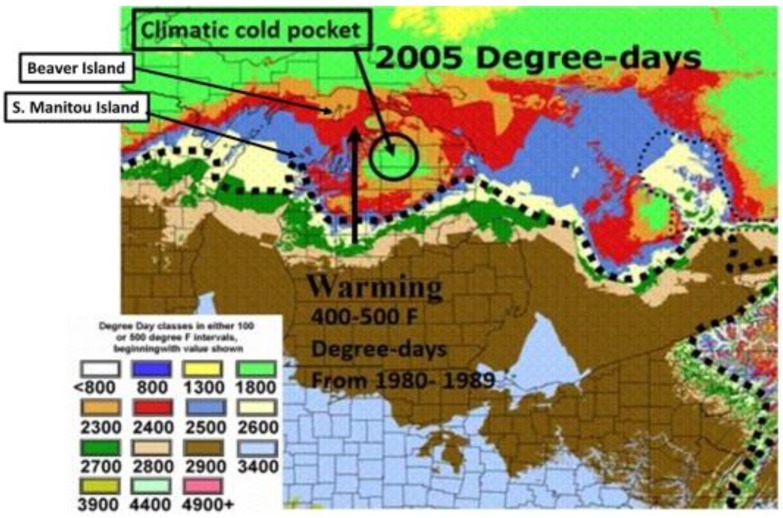
Enlargement of the thermal landscape in a warm Michigan summer (2005), showing D-days that are 500–600 °F, or more, greater than the 1980–1989 mean shown in Figure 3. Nonetheless, the “cold pocket” is still evident relative to the surrounding counties. The cold pocket has actually shown greater relative warming than these surrounding areas over the recent 2 decades (see Figure 7). Note that South Manitou Island in northern Lake Michigan has experienced greater warming than Beaver Island (and also Isle Royale island in Lake Superior), and introgressive traits from *P*. *glaucus* have been seen here (see text). The Degree-day values of color codes (Celsius) are shown in Figure 3 for comparison.

## 2. Biosystematic Levels (Bottom-Up Processes; Figure 1)

The classification of “subspecies” has enabled studies at the interface between systematics and population genetics, and represents a unit of biological organization that has been widely considered and used in conservation biology [30]. However, the geno-dynamics of populations need to become more broadly recognized, understood, and practiced across community [31,32] and landscape levels (e.g., “landscape genetics”[33,34,35]. Hybrids and hybrid zones represent unusually valuable “natural laboratories” that can be monitored for gene flow, introgression, ecological specialization, evolutionary divergence, reproductive isolating mechanisms, and hybrid speciation (and hybrid extinction). Hybridization and genetic introgression may enhance “adaptability” which is valuable in conservation translocations or restorations [16,17,36]. Perhaps, in the near future, specific genes conferring adaptation to specific climates, hosts, or environments [37] may be used rather than natural and human-induced hybrids. Nevertheless, such hybridization for genetic enrichment has been shown to be successful in fish [38] and endangered plants [39].

## 3. Ecosystems and Biodiversity Hotspots (Top Down Interpretations)

Global ecosystems have been mapped, showing heterogeneous species richness across the Earth, including “hotspots” with particularly high taxonomic species diversity of conservation value [40,41]. Understanding the historical climate [42,43] greatly helps in predicting genetic diversity of some hotspots [44,45]. Global warming has become a major concern for these hotspots because of ecological and genetic reshuffling at all levels (Figure 1; [4,11,12]). Interactions at the community level, population level, and individual level of ecological variation are fundamental to the maintenance of ecosystem hotspots of biodiversity worldwide. The underlying genetic diversity provides the building blocks of all biodiversity and this has become better recognized at each level (see Figure 1; [16,17,31,46]). However, in addition to the genetic, ecological, and biosystematics categories of life, effective conservation management programs must incorporate social, political, economic, and even religious factors at each location (Figure 1 [47,48]). 

It has become apparent that future lack of global sustainability will not likely be due to a lack of academic knowledge, but instead, due to unsustainable human behavior and socio-economic disincentives [49]. More ecologists need to expand their roles beyond their detached, objective technical skills and help integrate science into management, policy, and advocacy aspects [50]. This review recognizes the academic complexity of biosystematics categories and the arbitrary nature of socio-economic and/or political drivers [51,52], upon which many conservation management decisions are based. It will focus on insects (especially Lepidoptera), dealing with evolutionary diversity in nature conservation throughout current communities and the phylogenetic (historical) tree-of-life [53,54], with some additional emphasis on hybrid introgression and the enhanced genetic diversity it can generate for local adaptations, evolutionary divergence, and speciation. 

A switch in the units of conservation from the species level focus to intraspecific and interspecific diversity is beginning to take root scientifically with new genetic/genomic tools [33,55,56,57]. For example, higher levels of genetic diversity in a key species such as seagrass can enable ecosystem survival even under extreme climate stress [58,59]. Conversely, “genetic erosion” (inbreeding) can reduce genetic variance in populations and species and preclude adaptation to environmental stresses [60,61,62] and “speciation reversals” via genetic homogenization may be greatly catalyzed by loss of habitat diversity [63,64,65]. Hybrid introgression can serve as a useful source of genetic variation [21,66], especially when hybrids exhibit higher fitness than the parental species as in some novel or extreme environments [27,67,68,69]. Also, the preservation of phylogenetic diversity (evolutionary information) could enhance maintenance of rarity, species richness, functional diversity, and evolutionary potential [70,71], even in environmental specialists [72].

Most recent studies of environmental impacts on insect communities have been aimed at land use changes such as urbanization, agricultural intensification, habitat disruption and/or fragmentation, and only recently on impacts of rapid climate change [73,74,75,76,77,78,79,80]. A meta-analysis of 134 point source chemical pollution studies showed less effect on herbivores than their natural enemies, which may actually favor herbivores with the resulting enemy-free-space [81]. Insect pollinator, predator, and parasite communities provide fundamental services to forest and agricultural ecosystems, worth $8 billion per year to USA agriculture, and enhancing native flowering plants increases restoration of local biodiversity [82]. Climate change impacts on natural and agro-ecosystems have been reviewed recently [83,84,85,86,87,88] and the importance of all trophic levels (not a single species or crop alone) emerges as a central theme for future management [88]. While plant-pollinator interactions (=networks) have remained flexible even with some bee extinctions and pollinator quality decline over the past 120 years [89], landscape heterogeneity (physical/biological structure, including wild flower strips) as well as biotic composition will continue to be important for the climate-induced reshuffling of genes, genotypes, and species among multi-trophic-level ecosystem biodiversity ([73,82,90,91]; Figure 1). While evolutionary concepts have been fundamental to the science of conservation and biological invasions for decades, they have yet to be incorporated appropriately into management programs and policy [92,93,94]. Five major selection pressures on biodiversity (climate change, landscape disturbances, intensified agriculture, non-native “invasives”, and the spread of pathogens) cannot effectively be addressed when considered separately. Any one pressure can synergize, amplify, or buffer effects of another pressure, as described for pollinators [95].

Since many of the concepts discussed in this review deal with complex environmental impacts at many different levels of biodiversity (from genomes to current ecosystems and ancient phylogenies), the swallowtail butterflies provide an extensive geographical and historical data base, and will be used (Family: Papilionidae) as a focus and common thread (or “scientific basecamp”) for empirical excursions into these different issues in the next pages. The desired integration of behavioral, physiological, genetic and ecological aspects of recent climate-driven hybridization in both lab and field studies [96] has been possible with these *Papilio* [24,97]. With recently evolved sister species (*Papilio canadensis* and *P*. *glaucus*), their geographically extensive (and long-studied) hybrid zone, with hybrid recombinant “Late flight” hybrid populations (and likely incipient hybrid species), and with their very recently discovered “hybrid species, *P*. *appalachiensis* [26,27,99,100,101], we have a desired “speciation continuum” for research [102,103] with well-characterized natural and biogeographic histories.

The complexity of ecosystem responses, the lack of equilibrium conditions in communities and populations, combined with the recent rapid changes in environmental conditions (e.g., climate and pollution, and habitat destruction or fragmentation) are ultimately influenced by the underlying genetics (Figure 1). These communities, as well as that of “species”, subspecies, cryptic species, host races, or population [104], need to be integrated at all levels with ecological and evolutionary processes and conservation management considerations [34,35,105,106,107,108]. Understanding how climate change and landscape heterogeneity constrains or facilitates gene flow will certainly become a more important central focus for biodiversity conservation research in the future [109].

Landscape genetics may focus upon evolutionary questions about genetic isolation by distance or introgressive hybridization (*i.e.*, both physical and biotic landscapes [24,110,111,112]. It must also address ecological questions about landscapes, climates, different kinds of gene flow, local adaptations, and evolutionary divergence or convergence through time [64,114,115,116,117]. As discussed above, introgressive hybridization with gene flow between subspecies and between species can generate positive and negative results for conservation goals, and these need to be considered in designs of biological reserves and corridors [112,118]. Interspecific biotic interactions may significantly limit the range of species shifts and introgression at “hybrid zones” in the face of abiotic climate change [7].

Hybrid speciation [18,21,66,68,100,119,120,121,122] and speciation reversals [64,123,124] may both be generated by hybridization. With sexual selection, the hybridization may be unidirectional, with only one type of mtDNA usually of maternal origins [125], but see [126]. Ecological/behavioral reproductive isolating mechanisms may evolve faster on sex chromosomes than autosomes [127] with extensive implications for speciation. However, all of the genetic divergence and hybridization will have ripples of direct importance at other trophic levels in the ecosystem [31,128]. Complex interactions among population size, genetic variation, strength of selection, and gene flow for each population warrant individualized conservation management consideration [129].

## 4. What Should Be Conserved in Conservation?

A nicely summarized description of recent conservation activity is provided by Gillson *et al*. [93] “To date, the emphasis for conservation in a changing climate has been on expanding protected area networks to accommodate future climate space, based on range shifts predicted by bioclimatic species distribution models, together with the establishment and strengthening of habitat corridors and stepping stones to facilitate dispersal and migration” (see also [130]). However, a number of dynamic scenarios [80,87,88,131] and other ecological surprises have been encountered and must be dealt with [84,131,132]. For example, in addition to unequal phenological responses across trophic levels [132,133], different guilds of insects have different feeding adaptations and will exhibit different responses to environmental stress [134]. Warming may actually reduce the number of generations of some insect ectotherms [135,136], rather than increase the number of generations as generally expected [137,138,139]. Despite abundant examples of species range shifts upward in latitude and altitude with warming [79,131], there are some that lag or do the opposite [139].

If the goal is to make decisions and act “pre-crisis” rather than “post-crisis”, environmental managers will need both resilience in their ecosystems [33] and flexibility in their institutions [93,140] with limited budgets, abundant administrative rigidity, and momentum that is often in wrong directions. One new approach called “conciliation biology” [140] suggests that nonnative invasions may both hinder and help [141] management goals, and managed co-existence, rather than massive (and expensive) eradication attempts, might be the best solution. It is also noted that species ranges are very dynamic (rarely, if ever at equilibrium with climate) because of natural variance in abiotic factors, dispersal, disturbance, and various biotic selection forces. Therefore predicted climate shifts should not be the sole factor upon which to make conservation decisions. It has been pointed out that “conservation under current conditions is about managing change: retaining and restoring past community composition is no longer possible” [142].

Species richness and diversity are a dynamic balance between extinctions and creations. However, it has been estimated that of all the species that have ever lived on Earth, 99% are now extinct [143].The causes of and constraints upon novel genetic material [144] and genetic recombinations that facilitate evolutionary divergence may contribute in significant ways to the adaptability, resilience, and diversity enhancement of communities and populations that we wish to manage. The biological continuum has been observed to contain mosaic genomes [25,27,145], mosaic hybrid zones [104,146], across mosaic landscapes [31,73,147] that change via mosaic coevolutionary interactions [148]. The adaptive radiations of herbivorous insects and flowering plants have received considerable attention over the past half-century with their interactions suspected to be responsible for the high biodiversity they represent [149,150,151,152,153,154]. While the measurement and definitions of “specialist” (and generalists) varies across individuals, populations, communities, and through time/phylogenies [97,155], the role of specialists and generalists in the evolution and extinction of species seems fundamental [156,157,158,159,160].

## 5. Latitudinal Gradients in Global Biodiversity (Macroecological Patterns)

Perhaps the best known of ecological macro-patterns of diversity in nature are the latitudinal/altitudinal clines in species richness which usually decrease poleward and upward. Although some exceptions exist, this latitudinal diversity gradient (LDG) pattern (and altitudinal gradients [161,162]) has been observed in many plants, animals and invertebrates [163,164,165]. Many interacting hypotheses to explain this general pattern at a global level have been advanced in the past 5 decades, without a satisfying explanation [166,167,168]. Understanding latitudinal gradients requires knowledge on latitudinal range limits of species [169,170,171]. However, while major problems facing conservation of large vertebrates are habitat loss and genetic bottlenecks, we do not even know how many species of invertebrates and insects exist, let alone their distribution limits and sources of environmental stress [172,173].

In the face of climate warming, it has been suggested that the impacts for ectotherms will be more severe in the tropical (low-latitude) rather than temperate regions, because the magnitude of temperature increase is expected to push more tropical than temperate species outside their narrow “tolerance limits” [174,175,176,177,178,179,180]. Also it has been pointed out that levels of genetic variation in some tolerance traits are also lower for the tropics than in temperate regions [181] which may constrain adaptive potential to respond to climate changes [182]. Terrestrial ectotherms in the tropics are more limited than those in temperate regions in potential movement to escape climate change [183], and communities there are more vulnerable to disruption than those in temperate communities [184,185,186].

A recent review supports the view that biotic interactions in the tropics are basically much more dominant and important than at higher latitudes [187], but no single explanation of latitudinal gradients in global biodiversity suffices [188,189]. The high diversity of tropical plants [190] and host plant phytochemical specialization (narrow niches) and adaptive radiations of associated phytophagous insects has been a prominent candidate for consideration (“escape and radiate”) [149,151,158], however other factors such as historical climates and evolutionary phylogeny (historical host affiliations) [191] can play significant (but not independent) roles, as recently shown for the Papilionidae [192,193].

The original compilations of contemporary geographical distribution data and latitudinal clines in species richness for the swallowtail butterflies (Papilioinidae) supported the global LDG [194,195]. In addition, the role of feeding specialization (potential species packing and narrowed niches) was evaluated for all known (reported) host plants of these Papilionidae, and more host family specialists are currently found in tropical latitudes [97,196] with similar results seen for other Lepidoptera [197]. These data on latitudinal patterns of species richness and feeding specialization in Papilionidae have been subsequently used to examine phylogenetic patterns [198], raise conservation concerns [199], and develop historical biogeographic scenarios regarding recent glaciations [97,200,201] as well as evolutionary origins (phylogenetics) and ancient movement (phylogeogeography) of the swallowtails [193,202]. This historically-sensitive phylogenetic approach with the Papilionidae has provided the first analysis of multiple hypotheses shaping large-scale geographic patterns of species richness and diversification through time, from their origins more than 55 million years ago through major continental drifting and host plant shifting to the present [193]. One surprising result of these analyses [193] is that the warmer climate during the Eocene (56–36 mya) likely gave rise to a warm-adapted clade of the Papilionidae with highest species richness at high latitudes, which subsequently shifted to lower latitudes with cooling (36–24 mya), resulting in species richness that today is greatest at lower latitudes [195,196], possibly because of their current predominantly tropical host plant affiliations/distribution [97]. This latitudinal diversity gradient (LDG) scenario for the Papilionidae is contrary to the “out-of-the-tropics” hypothesis [189] (tropical origins and some recent shifts to higher latitudes, while also remaining tropical).

The “escape and radiate” hypothesis of host affiliations [149] is not incompatable with the “paleoclimates-enhanced rates of diversification” or cryptic plasticity [192,203] of Papilionidae, and host shifts from generalists to specialists [204] certainly provide opportunities for subsequent specialization and evolutionary divergence [158]. However, the concept that feeding specialization would likely lead to “evolutionary dead-ends” (jack-of-all-trades-master-of-none concept) [97,155,207] has been challenged recently, and an “oscillation hypothesis for speciation” has been erected [156]. It is pointed out that host plant range is dynamic between specialists and generalists [205], and new host-plants added to this range provide opportunity for “re-specialization”, divergence, and speciation [156,203]. Temperature-driven changes in host use [206] and genetic introgression [100] can create geographic range expansions for existing species (see [208]).

## 6. An Experimental Evaluation: Do Specialists Retain the Capacity for Generalization (Host Shifts)?

To evaluate whether the long-recognized feeding specialization and purported close coevolution [149] of selected Papilionid species on Rutaceae, Monimiaceae, Lauraceae, Magnoliaceae, and Annonaceae was an evolutionary dead-end, Scriber *et al*. [204] bioassayed larval feeding and survival abilities on a range of ancient Australian Angiosperms. The Lauraceae specialists *Papilio troilus* and *P*. *palamedes* were unable to feed on plants from any other family, and in fact had specialized on particular species within the Lauraceae to such an extent that abilities to use any other plant species were lacking were evident [97,209,210]. In contrast, all other specialist species assayed (*P*. *aegeus* on Rutaceae; *Graphium macleayanus moggona* on Monimiaceae; and *G*. *eurypylus* on Annonaceae; and the Magnoliaceae-specialized *P*. *glaucus* *australis* from southern Florida) were able to feed and grow on other plant families than their own, despite millions of years of host family specialization [204]. These findings illustrate the potential for long-recognized specialists to give rise to generalized feeding or host shifts as theorized in the “oscillation hypothesis” for speciation. Ability of such family-specialized insects to feed on these related ancient plant families may be due to a pleiotrophic cytochrome P-450 detoxification enzyme system for handing furanocoumarins in plants of the Rutaceae [151,211] that may be shared by *P*. *glaucus*, *P*. *aegeus*, and the two *Graphium* species (but not found in the *P*. *troilus or P*. *palamedes*; [212]). In North America, the host-specialized *P*. *palamedes* is experiencing serious threat to survival in the southeastern U.S. due to a geographically extensive plant pathogen infection that is destroying its primary (or only) host, red bay (*Persea:* Lauraceae) host plants [97,98].

## 7. Other Latitudinal Considerations

Latitudinal gradients in temperature certainly explain range limits of some species of insects, but unlike temperatures, the latitudinal gradient of seasonal changes in photoperiod is stable, and does not change with local or regional climate [213]. Asynchronous phenologies of different trophic levels (well-known from agricultural host-parasitoid interactions) can severely disrupt community interactions, as seen when insect post-diapause emergences track temperature but bud-break of their host plants depends on photoperiod photoperiod [214,215]. Invasive species predictions should include photoperiod as a consideration as well as other environmental factors because of differential impacts of photoperiods on diapause success from higher latitude European insect populations compared to lower latitude North American populations [213,216]. However, rapid genetic responses (evolutionary adaptations) in insects using photoperiod cues (daylengths [213]) have been reported as a result of earlier springs, later falls, longer summer growing seasons [217,218].

In summary, macro-ecological latitudinal patterns of global diversity (and hotspots) may be caused by diverse biotic and abiotic factors that interact with environmental changes [117,150,219,220]. It is important to remember that local community interactions (multiple trophic levels, abiotic stressors, and genetic variability) shape the local responses and capabilities for change that comprise the ecosystem [221,222]. Management of one species cannot be done without consideration of the whole system [105,106]. When trying to define and protect “biodiversity”, we need to always include underlying genetics (at all levels) as well as the overarching conservation issues of habitat destruction, disturbance, fragmentation, or pollution that may be more easily recognized. Historical climates and evolutionary phylogenies may also need to be included when planning for current or future management options.

For example, the entire ecosystem of communities can be impacted by invasive species [223] and the direct and indirect effects on ecological processes throughout the communities can be extensive [224,225,226,227]. Success of biological control agents (parasites, predators, and pathogens) depend on their synchrony host plants and herbivores [88,228,229,230,231]. At the community level, the success of invasive species in establishment depends on the potential enemy-free space (e.g., lack of pathogens [231] as well as herbivore genetic variation, and that of the natives [94,232,233,234]. Native insect herbivores will feed on non-native plant introductions in ways that have become largely predictable [235], but ecological reshuffling of community composition (Figure 1) may constrain the realized geographic distribution from its climate-predicted potential [230]. However, in Australia near Brisbane, the toxic introduced *Aristolochia elegans* from Brazil stimulates the endangered native Birdwing butterfly females to oviposit on its leaves (even though this plant species is lethal to its larvae). Nevertheless, recently, some of these butterfly populations showed signs of leaf detoxification, and larval consumption and growth and may be in the process of adapting to this plant [488].

Of more than 400 alien phytophagous insect species that have invaded North American forests [216,236], one of the most significant defoliating forest invaders in North America was the gypsy moth (GM), *Lymantria dispar* L. [52]. The devastation was not just from extensive defoliations, pesticide contamination, and (without pesticides) the resulting fecal and microbial contamination of some human water supplies. It was also was in part due to the various management programs which have caused significant non-target impacts throughout the ecosystem (including release 100 years ago of generalist parasitic biological control agents such as the *Compsilura concinnata* tachinid fly, which has been a major factor in the decline of native Lepidopterans such as the *Hyalophora cecropia* and the *Callosamia promethea* Saturniidae silkmoths [237,238]). The use of bacterial sprays (*Bacillus thuriengiensis kurstaki*) specific to Lepidoptera were safer for other insects such as pollinating bees, but under normal field conditions the pesticide (Btk residues) killed non-target Lepidoptera (*Papilio*) for 30–40 days post-spray [239]. Populations of endemic moths and butterflies were also impacted negatively in West Virginia and Virginia [240,241]. In addition, Redman and Scriber [242] found that gypsy moth defoliation significantly decreased growth and survival of native swallowtail butterflies, *Papilio canadensis* R & J near gypsy moth populations by reducing the host plant quality, and enhancing the parasitism rates in their larvae [225]. Similar results with various ecological ripples (top-down and bottom-up) have been seen in other forests and herbivores and illustrate that multiple biological levels will be involved [243]. “Extinction debts” in communities (e.g., from delayed impacts of habitat fragmentation or degradation [244]) can surface years later, making the ecologfical impact predictability of such habitat disturbances difficult [10]. In addition, management decisions are often paralyzed by additional political; economical, and social complications relating to “risk-perceptions” and economics [52,245], rather than made for sound ecological reasons.

## 8. Plant Genetics and Phenotypic Induction of Resistance

Hybrid poplars, experimentally grown for biomass production (at the NSF-LTER site) were in the path of expanding gypsy moth populations at the Kellogg Biological Station in southwest Michigan. Experiment supplementation of GM populations with egg masses added was conducted for several years to determine the differential roles that biotic (defoliation) and abiotic (fertilization) would have on the induced resistance (carbon-based phenolic glycosides; [246,247]) of these trees to herbivores. While hybrid poplars responded with phytochemical induction as predicted after defoliation [247], several different species of Salicaceae-adapted herbivores (including gypsy moths, *Lymantria dispar*; fall webworm, *Hyphantria cunea*; big poplar sphinx, *Pachyspsphinx modesta*) did not show growth and survival response differences among treatments (fertilizer+, defoliation+/fert−, defol+/fert+, defol−/or fert−, defol− [489]),but bioassays detecting subtle differences in levels of induced carbon-based defenses after defoliation with and without fertilization were possible using a lab-generated genetic continuum of hybrid and backcrossed species of swallowtail butterflies [248,249]. 

As with the other poplar-feeding insects, the *Papilio canadensis* larvae were naturally adapted to Salicaceae [246] and showed little difference in treatment responses; however, the southern sister species, *P*. *glaucus*, that prefers Magnoliaceae, all died on the hybrid Poplar (*Populus*) leaves of all treatments (again not permitting bioassay evaluation of treatment induction effects). We were able to hand pair the *Papilio* species and get hybrids and backcrosses with differing concentrations of detoxication enzymes [248] that consequently were differentially susceptible to the subtle induced phytochemical changes from defoliation and fertilization ameliorization [225,249]. This emphasizes how the genetic continuum from hybrids and backcrosse insects can extend into community interactions [250] and how the evolution of community structure and composition depends on evolutionary phylogenetics as well as phytochemical composition [251,252,253]. The ability of Salicaceae-specialists to switch to novel host plants with little or no phenolic glycosides [254] can help them to escape from various natural enemies that cue in on these allelochemicals [255], facilitating potential adaptive radiation. The continued ability to detoxify phenolic glycosides in Florida populations of *Papilio glaucus* [201] may represent ancient genetic polymorphisms (e.g., arising during the period when Florida was mostly under ocean water except for a few central ridge islands [201]) or pleiotrophic plasticity rather than a novel biochemical detoxification capacity from recent introgressive hybridization from older *P*. *canadensis* populations (harbored in the cooler refuges in the southern Appalachian mountains). While such introgression is recently increasing in the Great Lakes hybrid zone [24,150], it has not been seen in the northern Florida *Papilio* subspecies hybrid zone (Figure 2).

## 9. Genetic Variation across Species Ranges (Central-Marginal Hypotheses)

The diversity of species across latitudes will depend upon the range boundaries of individual species [24,131,172,256]. However, determining where these borders occur geographically for even a single is difficult because of the dynamic nature of its populations and abundance. Levels of speciation and adaptation often differ within a single species [117,254,257]. The determination of where species boundaries occur biologically is even more difficult to determine because of hybrid introgression, genetic porosity, and the blurring of taxonomic categories that include subspecies [30,258], host races [259,260,261], polymorphisms [262,263], cryptic species [101,264,265] and even “hybrid species” [26,27,68,69,100,101,120,121,266,267].

Species and subspecies boundaries have been recognized as very “porous” in vertebrates and invertebrates [25,30,268,269,270,271], and these classifications appear to be spread along as a continuum [104,272,273,274] in butterflies [24,26,274,275] and moths [276,277]. Although cryptic biodiversity may be lost with climate change [16], the evolutionary generation of genotypic novelties (or hybrids or new species) in the face of gene flow continues to enrich biodiversity in many taxa [21,55,56,103,278,279,280]. Rapid divergence of sex-linked genes contributes to reproductive isolation in tiger swallowtail butterflies [281]. Speciation often involves evolution of sex-linked genes as the driving force (as in mosquitos [282], which seems especially important when the female is the heterogenic sex [127,283,284], including hybrid speciation in butterflies [24,26] and birds [285].

As with biodiversity hotspots in ecosystems, so too genetic hotspots (e.g., some small subpopulations) may contain a disproportionate fraction of the genetic diversity [142,286]. Additional genetic hotspots include areas of interspecific [24] and inter-subspecific hybridization [30] as well as both “central and marginal” populations of the species’ range [131,142,287,288,289,290]. Genetic diversity does not always decline with outward distance from central range locations toward range margins in butterflies [291], and butterfly hybrid zones may have the highest diversity [24]. Even thermally-flexible species may be constrained in range movements with climate change, depending on constraints in their host plant switching potential [97,150,292]. Regarding range shifts in butterflies, it has been shown that ecological specialization often results in distribution declines, and also, poor dispersal abilities and large body size predisposes species to distributional decline [293]. Thus in the future butterfly communities may become more characterized by highly dispersive generalists [294].

Understanding the impacts of global climate change on genetic diversity within a species and within populations is essential to fully understand the global climate change (GCC) impact on biodiversity at all levels [16,17,33,169,295,296]. The parts of the geographic range to be targeted for conservation efforts in the face of climate change depends on the scale of species distributions [24,131,135], and the adaptations to marginal habitats [142,288], variation in central-marginal distribution of population densities [287], and their ecological dynamics [297]. The call for macroecology studies across the entire geographic range of a species can now be combined with genomic analyses of individuals and populations from the center and margins of the distribution [297,298,299] as well as across hybrid zones which may be mosaics themselves [104,146]. In this way, the benefits and risks of conservation decisions such as translocations can be assessed from a genetic perspective [15,300]. The historical component of genetic diversity within an evolving taxa can also now be assessed using phylogeography [193,301,302].

## 10. Climatic (Thermal) Extremes and Variability may Be More Important for Range Shifts than Mean Temperature Increases, (both in Winter and Summer)

Climate specialists with narrow tolerances for conditions associated with latitude and altitude, (e.g., humidity, mean temperatures, extreme temperatures, diurnal variation, rainfall, *etc*.) may arise due to ecological/evolutionary trade-offs (antagonistic plieotrophy [303]) or to DNA decay (accumulations of deleterious genes [304]), or because adaptation to the multidimentional “climate” components requires too many adaptation adjustments, or gets swamped by gene flow [305]. Thermal tolerances differ between marine and terrestrial ectotherms and potential ranges of terrestrial organisms are not realized in the tropical (equatorial) end of latitude ranges, perhaps due to increased biotic exclusion factors [187] or other differences with temperature variability on land [306,307]. The extremes of heat in summer can differentially impact insects such as hybridizing tiger swallowtail butterflies [308] and variability in summer/fall temperatures can have differential impacts on various traits, survival, or size as well [309,310,311,312,313,314]. Winter cold extremes and variability [315,316,317,318] can also exert strong selection on species range limits [319,320,321]. Mountain refuges may, however, be eliminated for some cold-adapted species with continued warming [163,322,323,324,325].

In addition to winter extremes and variability, another factor that can be important is the duration of these extreme winter cold stresses. For example, while we have seen that the geographic distribution limits of two hybridizing *Papilio* in North America has been relatively consistent for several decades [24,326], with their narrow zone of parapartry essentially delineated by voltinism constraints using thermal unit accumulations (Degree-days = 2,500–2,700 °F [326]) above their base developmental threshold of 10 °C (50 °F), the winter temperatures may also play an important role in permitting the northern *P*. *canadensis* and Late-flight recombinant hybrid genotypes to survive where the multivoltine *P*. *glaucus* cannot [327]. In addition to inability of the *P*. *glaucus* to complete a second generation north of the historical hybrid zone, even on the best host plants [328], the duration of winter cold extremes (18 °C, =0 °F; see below) may also be important in determining northern range limits for these *P*. *glaucus* (Figure 2). 

## 11. Background on Tiger Swallowtail Sister Species and Their Hybrids

In North America, there is an extensive plant transition zone (ecotone) from the Great Lakes region to the Appalachian Mountains and New England in North America that separates boreal and temperate deciduous forest biomes which also corresponds closely to the zone of hybrid interaction for many animals including insects (Figure 2 [328,329,330,331]). In the hybrid zone between recently evolved sister species *P*. *canadensis* and *P*. *glaucus* [332] the historical boundaries (1960–1997) of this North American hybrid zone are closely delineated by mean annual summer thermal accumulations (above a developmental base 50 °F, or 10 °C [328,333]). To the North, where it is cooler (<2,300 °F = 1,278 °C degree days; Figure 2 and Figure 3), *P*. *canadensis* is univoltine with a Z (=X)-linked obligate diapause and several other adaptations to allow it to successfully complete a full generation and survive all the way north to central Alaska [219,334,335,336]. To the south where it is warmer than 2,800 °F (=1,556 °C) degree days, *P*. *glaucus* is basically bivoltine (or trivoltine further south), with a facultative (photoperiodically induced) diapause [337,338]. However, recent warming has been extensive across the whole North American Great Lakes region from 1998–2012 (Figure 4).

**Figure 5:**
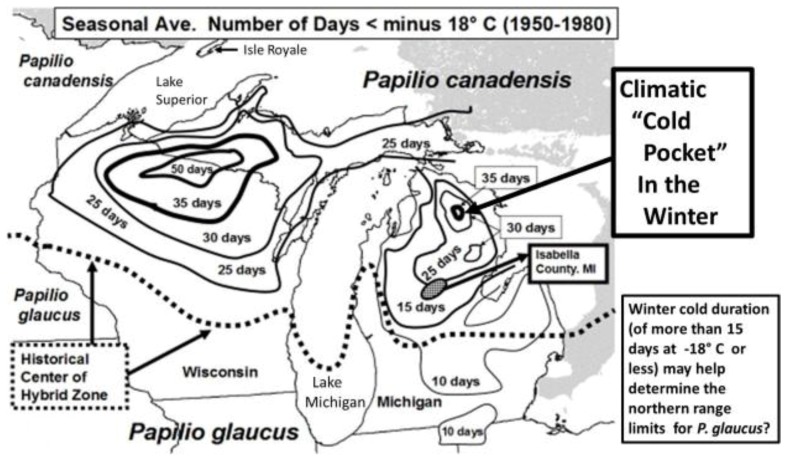
Climatic cold pockets during winter, showing the seasonal average number of days that were minus 18 °C or less (1950–1980) are indicated for Michigan and Wisconsin [339]. The historical hybrid zone between *P*. *canadensis* to the north and *P*. *glaucus* to the south is indicated by the dotted line. The *P*. *glaucus* have generally been found where fewer than 15 days with average temperatures at or lower than minus 18 °C occurred [326,346]. These cold pockets have shown winter warming trends during the past two decades (see Figure 9).

In Michigan and Wisconsin, seasonal isoclines with 15 (or more) total days annually of extreme cold stress in mid-winter (with temperatures reaching −18 °C; 0 °F, or less) closely correspond geographically to the northern limits of the historical range of *P*. *glaucus*. In contrast, *P*. *canadensis* exists where an average of 20–50 days with such temperatures occur (Figure 5). Short durations (4 days) of extreme warm and cold mid-winter stresses have been recently shown [327] to differentially impact diapausing pupae of these sister species of *Papilio* and their unique “late flight” interspecific recombinant hybrid (*LF*) populations [100,101]. The historical northern limits to the geographic range of *P*. *glaucus* in Michigan and Wisconsin is in fact correlated with the mean number of days with annual cold stress of minus 18 °C (0 °F), or colder (cf. Figure 2, Figure 3 and Figure 5). Pupae of *P*. *canadensis* in Alaska may regularly experience temperatures of −15 °C to −18 °C below the snow level [319,334]. In the absence of snow, the temperatures experienced in Michigan at ground level would also reach −18 °C for extended periods [339]. During the 1950–1980 decades, some parts of northern Michigan and Wisconsin annually average 50 days with temperatures at or below −18 °C (summer “cold pockets” in northern Michigan and its upper peninsula, where *P*. *canadensis* occurs; Figure 5). In contrast, southern Michigan generally experiences an average of only 5 to 10 days at this temperature where *P*. *glaucus* occurs.

## 12. Durations of Severe Mid-Winter Cold Stress

To evaluate ecological impacts of increased durations of cold stress exposure, we used split brood studies and simulated various mid-winter long term cold stress exposures times without snow cover (10-day, 20-day, 40-day, 60-day, and 70 days at continuous −18 °C) on diapausing pupae of larger, facultatively bivoltine *Papilio glaucus* (from Pennsylvania populations at 39° N latitude) and smaller, univoltine *P*. *canadensis* (from 43° N, in Vermont). We also included some *P*. *glaucus* pupae collected and reared at more subtropical latitudes in Georgia (at 34° N lat.) and a subset of interspecific LF hybrids (recombinant hybrid genotypes found on the cooler side of the thermally-defined hybrid zone at 43° North latitude in Vermont between *P*. *canadensis* and *P*. *glaucus* [100,340]. After mid-winter extreme cold stress durations were completed, pupae returned to “normal” winter conditions at 4 °C, until spring emergence at 22 °C and 18:6 h “long day” photoperiod.

The experimental mid-winter cold stress simulations (at −18 °C) of no snow cover were begun after two months of diapause under normal winter conditions (4 °C; of simulated conditions below snow cover). After various durations of mid-winter cold stress at −18 °C, pupae were returned to their normal winter storage conditions until spring emergences. In addition, groups of *P*. *canadensis* and *P*. *glaucus* pupae were exposed to 10 days and 20 days of cold stress, but at the end of their 6-month winter (instead of mid-winter at 3 months), simulating a late Spring freeze. Initial numbers of pupae available to distribute across treatments were: (*n =* 264 *P*. *canadensis*; *n =* 264 *P*. *glaucus* (PA); *n =* 87 *P*. *glaucus* (GA); and *n =* 89 “LF” hybrids). The 30-year average cold duration map of Michigan (−18 °C; during 1950–1980; Figure 5) was derived from the Climatic Atlas of Michigan [339]. A close correspondence of the northern limits for *P*. *glaucus* was noted where areas had fewer than 20 days annually reaching −18 °C. 

**Figure 6:**
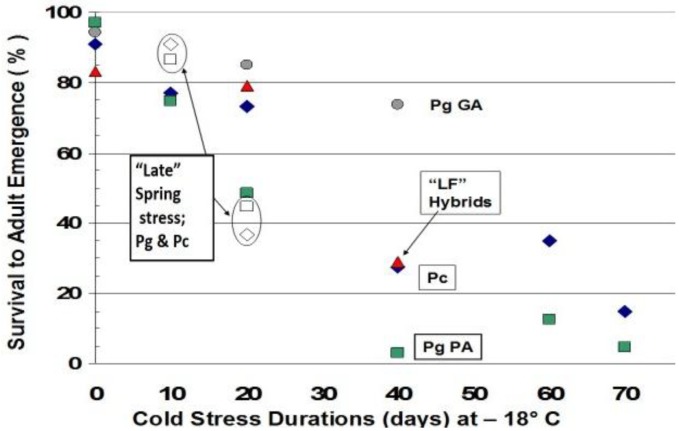
The pupal survival to adult emergences of *P*. *canadensis*, *P*. *glaucus* and Late-flight (LF) hybrids exposed at −18 °C for 10, 20, 40, 60, or 70 days in mid-winter. The normal winter control treatment (0 days at −18 °C) was at +4 °C in darkness. The number of survivors for each treatment shown here includes males and females together. Also, for comparison, a subset of these *P*. *g*. and *P*. *c*. were exposed to the cold stress just 10 or 20 days before being brought out of diapause in the late Spring (instead of mid-winter). Note that *P*. *canadensis* survival was better than *P*. *glaucus* at all durations greater than 20 days.

Negative impacts of mid-winter cold stress durations (at −18 °C) were evident as lower pupal survival for *P*. *glaucus* (PA) after 20, 40, 60, and 70 days (Figure 6). For example, after 40 days the survival of *P*. *glaucus* (PA) was only 3% and *P*. *canadensis* was 27.5%. At 60 days, survival was 12.5% compared to 35%, and after 70 days of cold stress, survival was 4.6% and 15% respectively (Figure 6). A normal winter (control conditions of 4 °C, for 6 months) for the late flight recombinant hybrid swarm individuals resulted in 17% mortality. However, LF survival after 20 days of cold stress was 79% and after 40 days was 29.1%, both of which were very similar to *P*. *canadensis* (Figure 6). Perhaps most unexpectedly, the *P*. *glaucus* pupae from Georgia had exceptionally high survival in spring emergences whether they had 20 or 40 days of cold stress (85% after 20 days, and 73.4% after 40 days at −18 °C; Figure 6). When 20-days exposure to the 4-day stress was at the end of winter, just before spring emergence, large negative impacts were seen in *P*. *canadensis* pupae compared to 20-days exposure in mid-winter (36.8% survival compared to 73.2% survival when stress was mid-winter), but not in *P*. *glaucus* (Figure 6), possibly due to their greater “depth” (intensity) of diapause [287]. Such differences might reflect the higher metabolic costs seen in these early-emerging obligately-diapausing northern *P*. *canadensis* and late-flight recombinant hybrid individuals [327]. 

In another comparative study, *P*. *glaucus* and *P*. *troilus* (Papilionidae) were less severely impacted by winter warming conditions than another butterfly, *Erynnis propertius* (Hesperiidae; [314]. Although all 3 species lost biomass during the winter diapause warmer winter simulations caused greater depletion of energy reserves in *E*. *propertius* than either *Papilio* species [314], (see also [292,311]). Unlike biomass loss in the hesperid, *E*. *propertius*, the biomass loss in *P*. *glaucus* and *P*. *canadensis* was not from desiccation, but instead by metabolism of dry weight biomass (not water loss, which appears tightly conserved in *Papilio* pupae; [327]). Of course the diapausing life stage of the insect may create different results, and the depth (or intensity) of diapause varies geographically, as do thermal performances of other insect populations [33,101,135,175].

The assumption that high latitude insect species generally have broader tolerances to thermal variation than more tropical species [174,175,305] has not been thoroughly evaluated. Alternation of thermal regimes (repeated chilling and warming) may help break (or alter the depth/intensity of) the prolonged diapause of some insect species [341]. Our work with the North American high latitude, univoltine *Papilio canadensis* R and J and the lower latitude, bivoltine *P*. *glaucus* L. tiger swallowtail butterflies has recently shown the opposite; when the thermal variance was imposed during mid-winter, the diapausing pupae of northern *P*. *canadensis* were more susceptible and experienced increased metabolic costs compared to *P*. *glaucus* [327]. However, as we show here, when the extreme cold stress (−18 °C) durations are constant, the high latitude *P*. *canadensis* does survive better than the lower latitude *P*. *glaucus* (from PA) at all durations of 20 days or more under cold stress (Figure 6). The diapause depth is deeper for *P*. *glaucus* than *P*. *canadensis* (and also for LF hybrids; [492]), and Georgia pupae apparently have even deeper diapause than those *P*. *glaucus* from Pennsylvania near the hybrid zone (Figure 2), presumably preventing extra metabolic costs required for early emergence under seasonal thermal constraints. Latitudinal differences in depth (or intensity) of diapause are known for insects [342,343,344]. 

## 13. How Fast Do Insects Respond with Size Increases to Local Warming?

**Figure 7:**
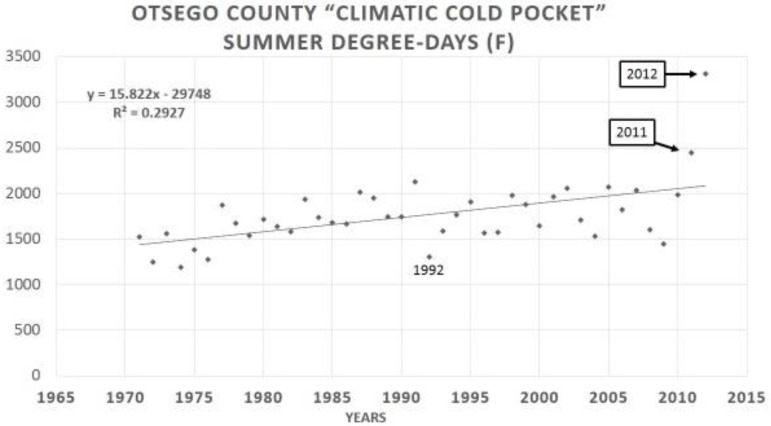
The mean Degree-day accumulations (F° = 9/5 C° for temperature conversion factor) for the Otsego cold pocket during the 4-decade period from 1971–2012. Note the “cool” 1992 summer [463], and the exceptionally warm 2011 and 2012 summers. Regression is significant at *p* = 0.01 level (R² = 0.293).

**Figure 8:**
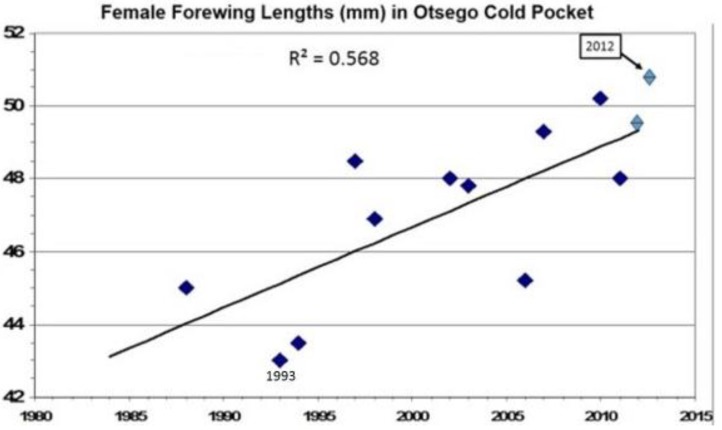
The annual mean forewing lengths of female *P*. *canadensis* (*n =* 125 in 13 years) captured in Otsego County, Michigan cold pocket. Note that the 1993 and 1994 small sizes follow the cold 1992 summer (Figure 7). (Regression is significant at 0.01 level; R² = 0.568).

Severe summer degree-day constraints on development of *P*. *canadensis* has resulted in smaller females in Alaska and in northern Michigan “climatic cold pockets” due to insufficient time to grow larger [219,335,336] than at lower latitudes (see also these opposite Bergmann’s rule trends for males [345]. However, during the past 15 years of steady and rapid regional warming in North America around the Great Lakes, there has been significant summer warming, reflected by annual summer Degree-day accumulations especially in these Michigan cold pockets (Figure 7), with a correspondingly significant increase in female size (as indicated by forewing lengths; Figure 8). This timing corresponds with known hybridization [346] and extensive introgression of many *P*. *glaucus* traits northward across the historical hybrid zone in Michigan (reviewed by Scriber [24]).

**Figure 9:**
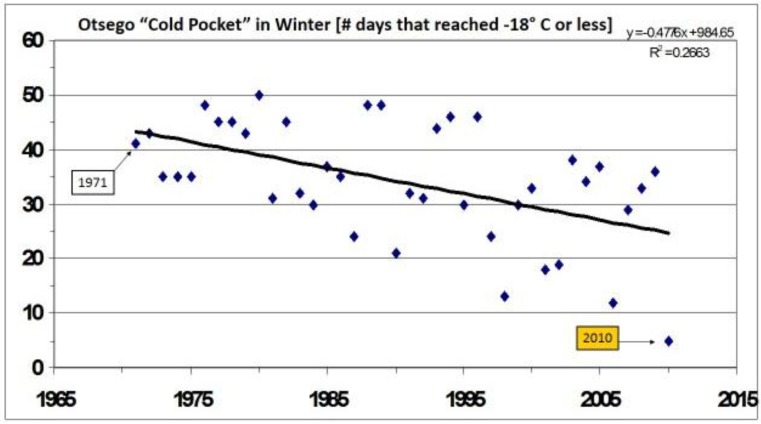
The number of winter days reaching −18 °C (or lower) in the climatic cold pocket [383]. (R² = 0.266, *p* = 0.01).

**Figure 10:**
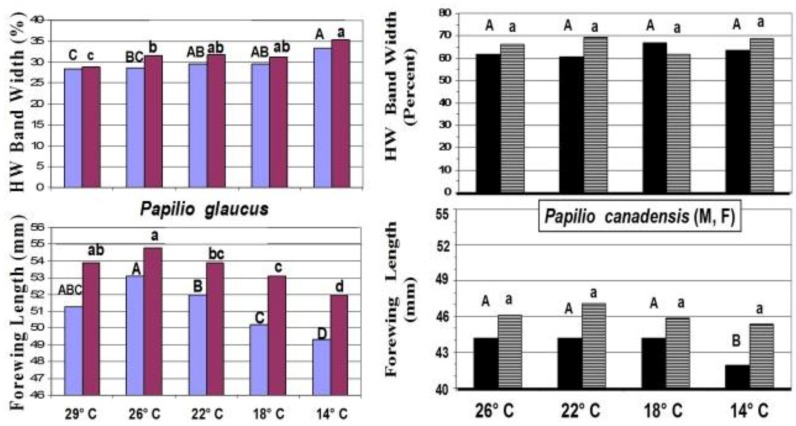
The forewing lengths and relative hindwing black band widths of the anal cell in; (**left**) *P*. *glaucus*; and (**right**) *P*. *canadensis* as a function of Spring emergence temperatures (under 18:6 h photoperiod). Males are on the left (blue, solid black), females on the right (red, grey stripes). Significant differences between treatment means are indicated by different letters, by sex.

However, rather than genetic introgression from the larger southern sister species (or a microevolutionary adaptation), these female size increases may simply represent a phenotypically plastic response to the release from summer developmental constraints in this hybrid zone [328]. It is also noteworthy that the period of extreme cold stress (number of days at −18 °C or less) in the Otsego cold pocket has declined steadily over these three decades (Figure 9). The increase in size of females in the warming “cold pocket” area could also reflect a reduction in metabolic expenditures for diapausing *P*. *canadensis* pupae during the warmer winters and springs. However, our studies in controlled environment chambers showed significant reduction in forewing lengths of *P*. *glaucus*, but not *P*. *canadensis* (Figure 10a,b), thus suggesting phenotypic flexibility or genetic introgression as most likely explanations.

A key point is that insects have many possible ways (genetic adaptations and phenotypic plasticity) to adjust to extremes and variation in temperature stress during both summer and winter, but adaptations across all life stages [334] should be considered (and across different seasons) to understand the relative contributions from genetic and phenotypically-based adaptations. Changing environments will be met with genetic and non-genetic responses in organisms [347,348,349], and impacts on diversification and speciation may be considerable [182,350]. Temperature stresses can also affect courtship genes, cytonuclear interactions impacting fertility and mortality in insects, as well as speciation genes [351]. 

## 14. Species level “Invasive Genes” (Genetic Introgression)

“Invasive genes” (into new species and populations) need to be recognized and addressed in management of conservation programs as much as invasive species into communities. Hybrid introgression may represent an invasion of genomes [274] which may create “hopeless monsters”, or transgressive phenotypes beyond those of parents (with sterile and/or inviable phenotypes [123]). On the other hand, such hybrids might also represent a mechanism for “hopeful monsters” with fitness greater than either parent [122,353], and which may even be useful in conservation management as a type of “species rescue” [354]. Climate-related traits can evolve rapidly in plants and animals [355,356]. Hybridization of two endangered (but very closely related) manzanita plant species localized near San Francisco (*Arctostphhylos montana*, and *A*. *franciscana)* has resulted in controversy regarding “pure” species status and the protection under the law. Recovery efforts (and endangered species status) began in 1979 for Ravens manzanita, and yet no natural seedling establishment is known, and recovery sufficient to justify delisting may not be possible without natural wildfires, prevented in San Francisco [357,358]. The rare Franciscan manzanita may be a hybrid with similar difficulties as the Ravcens.

Whether adaptations can be fast enough to adjust to rapid change depends on several factors [359,360,361]. Hybridization might allow exchange of adaptive genetic biodiversity between existing species as well as potentially generating hybrid species [21]. In order for this approach to work best, it has been suggested that assortative mating in the field should be intermediate to allow hybridization, but to also enough maintain separate species [354]. If hybrid inferiorities (outbreeding depression) do not result, this introgressive hybridization (as between sister species) provides one method that may be rapid enough in its generation of novel genetic diversity to keep up with rapid shifts in environmental factors such as climate change [66,119,129]. The management capacity to drive transgenes into natural populations of pests or disease vectoring insects for genetically modified attenuation of pest damage or danger, may also be a feasible genetic management tactic [362].

The swallowtail butterflies show extensive natural hybridization [272,332,363], genetic introgression [25], evolutionary divergence with gene flow inside the hybrid zone [24] and illustrate temporal reproductive isolation and hybrid speciation [26,100,101]. The use of this species group [272,364,365] as a model species is facilitated by the capability of these species to be hand-paired in the lab to produce interspecific hybrids and backcrosses for bioassays of oviposition preferences, larval survival and growth rates, and pupal diapause genetics [219,265,309,310,311,366,367]. Interspecific matings in the field show an asymmetrical preference where wild males of *P*. *canadensis* as well as wild males of *P*. *glaucus* both prefer size-matched females of *P*. *glaucus* when tethered next to each other [368,369]. Also, because *P*. *glaucus* and *P*. *canadensis* females and males are multiple maters (polygynous and polyandrous [370]) the possibility exists for the production of individuals of a “pure parental type species” and interspecific hybrids from a single female [371]. Divergence and hybrid speciation with gene flow has been possible to observe in these sister species because they are closely enough related to produce the entire spectrum of recombinant genotypes to hybrid species (*P*. *appalachiensis* [26,99,332]) to regular species, such as *P*. *glaucus* and *P*. *canadensis* [332]. Globally, this appears to be the case for many Papilionidae species [367,372]

## 15. The “Hybrids Are Bad” Concept

Inbreeding-stress interactions have been shown in meta-analyses to reduce fitness relative to outbred individuals (with “inbreeding depression” increasing with increased levels of stress [60,373]). Complications involved in understanding inbreeding stress interactions and impacts on local populations and biodiversity at other levels await additional studies, which should include natural populations in the field [62]. Genetically impoverished populations have reduced fitness, diminished disease resistance, and lower evolutionary potential [374,375], and individuals that are inbred show accelerated frequency of extinctions [376,377,378]. However, outbreeding depression can also occur [379].

The “tension zone” type of hybrid zones assumes stability is due to an equilibrium between selection and dispersal, but dispersal is dynamic and hard to estimate accurately [380,381]. Some maintain that hybrids and/or hybrid introgression are ecologically unhealthy and evolutionarily mal-adapted, contributing to population declines and/or extinctions as “mergers” or de-speciation, of other “good” species [29,33,62,64,65,120,123,124,382,383,384,385,386,387]. Invasive species may lead to extensive introgression of neutral genes [388]. Introgression usually occurs more from local to invading species [388], as is the case of range expansions [389]. However, it can also go from the native into the invading species. Hybrid zones themselves can be maintained by abiotic (exogenous) selection factors and have sometimes been assumed to be constrained by environmental boundaries and unlikely to move much. However, moving hybrid zones have become much more evident lately [380,388] with some movement recently driven by regional climate warming as seen with *Papilio* sister species [24].

These prevailing ideas that hybrids are “evolutionary dead ends” and always less fit than parental species [123] have also been disproven recently with several examples of introgression, evolutionary divergence, and homoploid hybrid speciation [21,26,66] (see also section below). In addition, natural hybridization can lead to greater gene dispersal, higher genetic diversity in populations, and, in some cases, improved fitness locally [67,355,390]. 

## 16. Plant Hybrids, Hybrid Zones, and Their Communities Should Be Protected (Community Genetics)

Plant hybridization is extremely important for plant evolution [391] and for dependent arthropods species [392]. The natural hybridization in North American Salicaceae (poplars, *Populus* spp.; and willows, *Salix* spp.) and Tasmanian *Eucalyptus* spp. has been shown to have valuable and extensive roles in maintaining diversity (taxonomic, genetic, and phytochemical) in plants and the animals on these hybrids or even in their aquatic and terrestrial communities (including, fungi, understory plants, vertebrates as well as arthropods: [31,393,394,395,396,397,398,399,400,401,402,403,404,405,406,407]. Similar community differences in plant hybrids in *Quercus* [408] and may be due to the plant phytochemistry and/or previous defoliation status (*Populus*; [249]). It has been shown [409] that plant hybid zones may act as “sinks” for pests (see also [396]), and also as “bridges” across parental host plants [395]. However, it is also known that hybrid plants may be more resistant than either parental species, as shaped by genotype/environmental interactions [397,410,411,412,413,414] and all plants show seasonal declines or increases of key allelochemicals, nutrients, and minerals [415,416]. Recombinant hybrids (in *Populus* spp.) retain heterozygosity at many loci, with implications for reproductive isolation [417]. Hybridization in peripheral populations of rare ploant speciues can provide novel diversity and adaptive potential, and such sites/hybrid populations should be conserved [288,289,290].

## 17. Global Change Impacts on Immune Functions and Disease Resistance (Are Animal Hybrids More Resistant to Disease?)

Climate change has resulted in large-scale infectious diseases in plants, animals, and humans [418]. Pollutants (chemical contaminants, including pesticides) can impact ecosystems directly and indirectly, via altered host-parasite or pathogen interactions, and invasive species may impose stress on native species or enable transmission of parasites [419,420,421]. Global temperature variation can also stress organisms and alter immune functions which contribute to some species extirpations [422]. As mentioned above, such stress can be catalyzed by a narrow genetic base in some (inbred) individuals [62,378]. Allozyme heterozygosity-fitness (growth, fecundity, survival, *etc*.) correlations show an overall increase in fitness with increased genetic heterozygosis [423] for various species [424,425]. However, the generality of these patterns has not been elucidated clearly and heterozygosity at microsatellite loci is not correlated with fitness as were allozymes [426]. Although “hybrid vigor” may contribute to resistance against parasites in interspecific crosses of mice species [414], virtually nothing is known about hybrid insects (relative to parental species) with regard to resistance to parasites and pathogens [353,398,413,427]. Nonetheless, the role of disease ecology in conservation may become more prominent as a driver of community responses with climate change [77,428], especially with the loss of biodiversity [418]. Migrating insects (and possibly other animals) have lower protrozoan parasite loads than the non-migrating populations (such as monarch butterflies that stay north during the milder winters, rather than fly to Mexico [418,420]). In addition to escaping infected habitats (migratory escape), some culling of diseased individuals may occur during long migrations (migratory culling [421]).

Problems identifying introgression and hybrids classes (e.g., F-1, F-2, backcrosses, *etc*.) makes generalization about “hybrid fitness” difficult [353,413]. Hybrid mice have been shown to have more parasites than parents [429]. Hybrid pocket gophers may be resistant to chewing lice that track parental genotypes [430], and hybrid (Africanized) honey bees may show differential resistance to some parasites such as *Varroa* mites [431]. In our 3-decades of hand-paired hybridization of swallowtail butterflies, we have reared many thousands of larvae on various host plant species [206,330,367,432]. In some thermal regimes and on many host plant species we consistently found clear evidence of hybrid vigor with more eggs produced, more neonate larvae, faster growth rates and durations from neonate larvae to the pupal stage, and larger pupae (see Table 1). In addition, our F-1 hybrids (and most backcross larvae) reared in controlled environment chambers appear to be clearly more resistant to the unknown pathogens that have largely wiped-out the contemporary parental species of *Papilio* for the past 2 decades. The 2-year average of neonate to pupal survival was 60%–65% for both of the reciprocal hybrid genotypes, while greater mortality (disease) reduced overall survival to 15% to 38% for the parental species for two hostplants. On the other hand, the preferred host for both parental species *P*. *glaucus*. and *P*. *canadensis*) is black cherry, *Prunus serotina* Ehrh., showed significantly more mortality (sick and dying in the 4th and 5th instar larvae) for the parental species than either of the reciprocal hybrids (Table 1). Similar results were seen for both parental species on tulip tree (*Liriodendron tulipifera*; Magnoliaceae) at 15, 23, and 30 °C [433]. The fact that any survival occurred for the *P*. *canadensis* on tulip tree may be due to subtle genetic introgression into some of the populations sampled by Donovan and Scriber in 1999–2000 [346], which was when extensive autosomal Salicaceae detoxification abilities were seen moving northward across most of Michigan [150]. Similar concern about “invasion genetics” and hybrid *Rhagoletis (*fruitfly) species in Europe include potential phenology and host shifts that could result in new hybrid pests with new host preferences as seen in other *Rhagoletis* species [434].

**Table 1:** Hybrid vigor in swallowtail butterfly sister species. Data from Scriber lab 1982–2003. Significant differences indicated by different letters at the *p* = 0.05 level (Tukey’s tests). Controlled environment chambers at 22–23 °C and 16:8 h photoperiod.

Species/genotypes	Mothers	Mean total Eggs	Mean Viable Eggs,%	Total Duration neonate-pupal stage (days)	Mean Pupal Fresh Weight (mg)	Growth Rate Mg/day
*Papilio glaucus*	246	110.5 b	65.3 ab	32.5 bc	927 b	28.5 c
*P. c.* × *P. g.*	17	77.1 c	68.0 a	26.2 a	1009 a	38.5 a
*P. g.* × *P. c.*	73	167.1 a	62.3 b	30.6 b	1089 a	35.6 b
*Papilio canadensis*	305	53.5 d	58.9 c	34.1 c	752 c	22.0 d

The vigorous growth of hybrids apparently reflects enhanced resistance to these unknown laboratory pathogens, but we were unable to confirm either bacterial, viral, fungal, or microsporidians as primary causes [490]. It would be beneficial to study the differential susceptibility to these various natural pathogens (*Serratia*, *Nosema*, *Entomophaga*, *etc*.) of herbivore hybrids and parental species. It would also be informative to add backcrosses at different temperatures and on selectively stressful marginal and favorite hostplants could provide a valuable base for predictions of insect/plant/microbe interactions under climate change. 

Our *Papilio* *glaucus* and *P*.* canadensis* appear to be *Wolbachia-* free [491], however, *Wolbachia* infections (a maternally-inherited endosymbiont bacteria) have been reported in as much as 65% of invertebrates tested so far with detrimental effects on the populations [435,436]. The introgressive hybridization associated with *Wolbachia* parasites and hitch-hiking mitochondrial DNA may result in widespread selective sweeps with infections crossing species boundaries [436]. The potential implications for Lepidoptera are serious. 

Small changes in the thermal environment (e.g., host body temperature, or “fever” and ambient temperatures in both ectotherms and endotherms) can have significant impacts on the resistance and recovery of diseased or parasitized hosts as well as pathogen virulence, but more studies are needed [437,438]. The dynamics of disease and parasite interactions with insects under climate change and also for human health issues, looms as critically important area for research [418,419]. The costs of immune response to parasitism can reduce resistance of insects to starvation, desiccation, and other stresses [439] and the immune responses of specialist and generalist insect herbivores may differ with host plant chemistry [440]. While elevated temperatures (fever) help some insects, some parasites actually alter the insects’ behavioral responses such as thermal preferences that favor the parasite not the host insect [441]. Some diseases may actually be facilitated by hybridization of the pathogens themselves [442,443]. All of these once again highlight the need for comprehensive integration across all levels of biological organization (Figure 1).

## 18. Translocations (and Assisted Migration) in Changing Environments for Maintaining Evolutionary Potential and “Genetic Rescue”

Recently rapid but more predictable rates of environmental change have made “translocations” of rare, restricted, and genetically-impoverished species a conservation tool to consider more seriously [300]. Ecological restoration (with biodiversity corridors or “biolinks”) includes community diversity and functionality, while conservation translocations (genetic capture, genetic rescue, genetic restoration and genetic adaptation) are aimed at populations of a single species. Success enhancing abundance, resilience, and persistence, depends on the community interactions as well as the genetic diversity, plasticity and local adaptations [300,444,445]. While translocations may provide a type of insurance against future climate or other environmental changes, cases need to have risks evaluated individually [300,446,447,448], and cooperation of resource-managers and scientists works best [448].

For example, moving some individuals from warm-adapted populations to colder locations (assisted colonization) may facilitate adaptations of cold-adapted populations for future warming climates with minimal ecological risks [33,449]. Experimental translocations [292,450] and lab studies [142,308,327,334] can help clarify some factors that constrain local adaptation and shape range limits. Migration is another form of adaptive movement or annual translocation that depends on coordination of seasonal growth, physiology, and reproductive synchrony, as is the case with diapause [451,452]. Different habitats for feeding and breeding of monarch butterflies are threatened by forest and farm habitat destruction and possibility of severe weather [453]. Photoperiod changes provide a more stable environmental cue over latitude than changes in local or regional climates, and are involved in shaping the ranges of many native and invasive species [213].

## 19. Are Locally-Adapted “Specialists” (or Local Endemics) More Vulnerable to Climate Change or Conservation Translocations (Assisted Colonization) than “Generalists”?

While host plant shifts to exotic plants can lead to speciation [454], rapid morphological adaptations [455,456], and escape from natural enemies [457], they can also play an important role in the “oscillation hypothesis” of evolutionary diversification [97,156]. Geographically widespread insect herbivore generalists show a greater propensity for use of novel, exotic hosts than geographically constrained specialists [458,459], and their chances of extinction are less. Local host specialists [150,257] in parts of the herbivore species range can, however, diverge genetically and become “cryptic species” as with the red-pine shoot moth, *Diaryctria resinosella* [460] and with the Tasmanian butterfly *Graphium macleayanum moggana*, a specialist on a single plant species, southern sassafras, *Antherosperma* *moshatum* (Family: Monimiaceae [461]).

Local endemics may be the groups in greatest need for translocation management decisions in the face of continuing rapid climate changes [261]. Islands often provide such evolutionary hangouts for unique local genotypes and species, but the biotic and abiotic forces may disrupt the stability of these small isolated populations and render them vulnerable [92,169]. This is perhaps why the greatest number of Papilionidae species currently under threat are on islands [199]. In northern Lake Superior (Isle Royale) and Lake Michigan, the *P*. *canadensis* appear relatively “pure” with little introgression from *P*. *glaucus*. Despite its high latitude at 45° N relative to the historical hybrid zone in Michigan (Figure 2 and Figure 3), South Manitou Island has been shown to be a consistent refuge for recombinant hybrid genotypes since at least 1991 [335,462]. This island population has hybrid-like diagnostic traits for morphology (e.g., hindwing black bandwidth; see below) and Z-linked introgression [24]. Again, thermal accumulations in warm years (as in 2005; Figure 4) might explain this introgression from the southern *P*. *glaucus*, but the island may also provide a northern “refugium” for these unique recombinant genotypes during cool years.

## 20. Rapid Genetic Changes (Evolution)

Rapid phenotypic responses to environmental change have been reported [217,218,355,356,456], but it is not always clear how much of these are genetically-controlled or phenotypically plastic. One example is body size, which has many ecological fitness implications (see review [345]). Many responses attributed to adaptations under changing environmental conditions may simply be environmentally-induced plastic responses rather than micro-evolutionary adaptations [182]. 

The forewing lengths of female *Papilio* from the Otsego “climatic cold pocket” show significant increases during the past 15 years, which correlate with more summer degree-days accumulated (Figure 7). Such rapid phenotypic responses may be due to size constraints on voltinism interacting with host plant nutritional quality [328,336] For example, smaller females continued for 2 years after the cold 1992 summer (Figure 7 and Figure 8; [463]). However, it is not clear if this may be due to longer time for summer growth after being released from seasonal constraints on size [334,335], or due to milder winters which require less metabolic expenditure for overwintering pupae (Figure 9, with −18 °C days declining over the 15 years), or both. Northward introgression of *P*. *glaucus* traits [346] could also be contributing to larger sizes locally [24]. While rapid evolution is possible with introgression, phenotypic plasticity seems more likely here, although “adaptive phenotypic plasticity” can lead to genetic differentiation and speciation [464].

## 21. How Fast Can Animal Speciation Be?

We cannot increase “species richness” without more (or new) species. However, speciation is usually thought of as being relatively slow (millions of years). The origins of the Papilionidae family of swallowtail butterflies was 52 milion years ago in the Eocene, and the *Pterourus* clade (containg the *P. glaucus* group) was 24 mya in the Miocene [465]. Even when speciation is relatively rapid as with *P*. *glaucus* and *P*. *canadensis* (Kunte *et al*. [26] estimated divergence times of 600, 000 years ago), such divergence and speciation is clearly not rapid enough to be part of any contemporary management plans. However, homoploid hybrid speciation can be much faster, possibly only a few dozen generations [267,340,466,467], sometimes with multiple independent origins, as seen in butterflies in extreme environments [69,468]. Can such rapid homoploid hybrid speciation rates be enhanced? There are genetic complexities involving reproductive isolation and several ecological explanations for why divergent natural selection initiates the speciation process but does not finish it [468,469]. For example, much evidence exists for thermally-driven divergence in adaptations, but little evidence exists that these lead to speciation [179,278].

Taxonomic insect groups such as *Papilio* have traditionally been identified using morphological criteria [470], and unlike the case in some groups, the tiger swallowtail butterflies of the *P*. *glaucus* group also show concordant phylogenetic trees for allozymes [363,364], mt DNA [272,283] and AFLPs [26]. However, despite recommended designation of evolutionary significant unit (ESU’s: [471]), mt-DNA does not always match the biotic discontinuities or “species” designations [472,473], and because of these difficulties delineating species (physiological, theoretical, empirical, and definitional [104,274,275,473,474,475,476,477,478]), it is recommended that conservation biologists use more quantitative descriptions of variation within and among clusters of organisms that the traditional “species paradigm” [473,475]. Studies of speciation could best be framed as studies of divergence in genotypes and phenotypes and the origin of reproductive isolation over time [70,104,476,478,479,480]. However, common focus and recurring emphasis on the reticulate nature of speciation, often fails to give attention to the increasingly important recombinant homoploid hybrid speciation in animals [69,120,121,266,267,467]. The time frame for such speciation events may be much shorter than traditionally presumed.

## 22. Rapid Hybrid Speciation in Recombinant *Papilio* Hybrids Seems Feasible with Post-Diapause Developmental Delays and Temporal Reproductive Isolation

**Figure 11:**
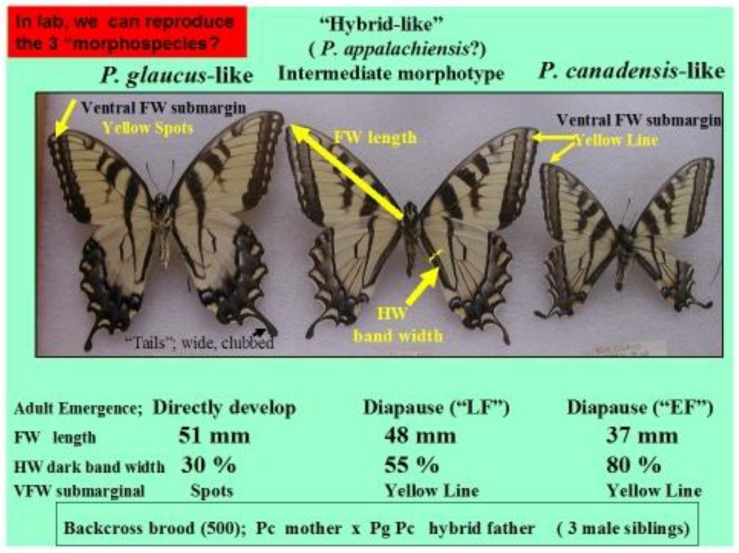
The basic morphospecies of *P*. *glaucus* (multivoltine), *P*. *appalachiensis* (univoltine delayed emergence), and *P*. *canadensis* (inivoltine) showing species-diagnostic traits (FW length, HW band width, and ventral submarginal spots/band [99,330]). These 3 individual phenotypes are genetically distinct siblings that were produced by hand-pairings in the lab (a backcross of a *P*. *canadensis* mother with a hybrid male). In addition to morphometric segregation with diapause regulation [481] (Figure 12), additional backcrosses of hybrid *P*. *glaucus* and *P*. *canadensis* to a parental species also resulted in high levels of recombination [10] and recombinant genetic segregation of key Z (=X)-linked ecological traits [24,482].

**Figure 12:**
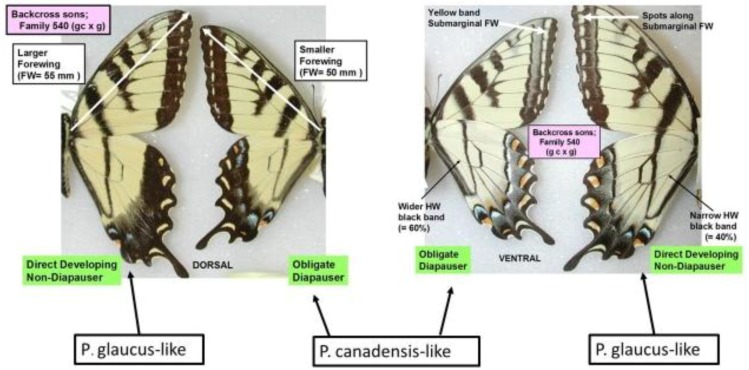
Typical wing patterns of backcross sons (siblings) showing segregation of *P*. *canadensis*-like traits (e.g., relatively wider hindwing band widths in the anal cell) with diapause and *P*. *glaucus*-like traits with direct development. The z-linked *P*. *glaucus*-like Ldh-100 allozyme is closely linked with the od- gene for facultative diapause (95% of females that directly developed), while the *P*. *canadensis*-like Ldh-80 and Ldh-40 alleles were linked with the obligate diapause gene (od+) [24,482]).

While our estimates of divergence of the hybrid species mountain swallowtail *[Pterourus (=Papilio) appalachiensis]* hybrid species from both parental species, the northern *P*. *canadensis* and the southern *P*. *glaucus*, was only 100,000 years ago [26]. In only 3 generations with a lab hybridization and a backcross, we are able to produce all three morphospecies (*P*. *glaucus*, *P*. *canadensis*, and *P*. *appalachiensis*; Figure 11) in siblings. Furthermore, the *P*. *glaucus* morphotypes directly developed in the lab under long day photoperiods, while the univoltine *P*. *canadensis*-like and *P*. *appalachiensis*-like individuals diapaused under the same long-day (18:6 h) photoperiod conditions, apparently having the Z-linked obligate diapause gene (od+) [24]. With post-diapause delays, the *P*. *appalachiensis*-like morphospecies would also immediately be reproductively isolated (temporally) from the flights of both parent type species. We have seen similar morphological and physiological divergence with the late-flying incipient species (natural homoploid hybrids) under field conditions inside the hybrid zone in Vermont [101]. In laboratory backcrosses, the species diagnostic morphological features consistently segregate out in these hybrid offspring uniquely with direct developers versus diapausers [481] (Figure 12).

**Figure 13:**
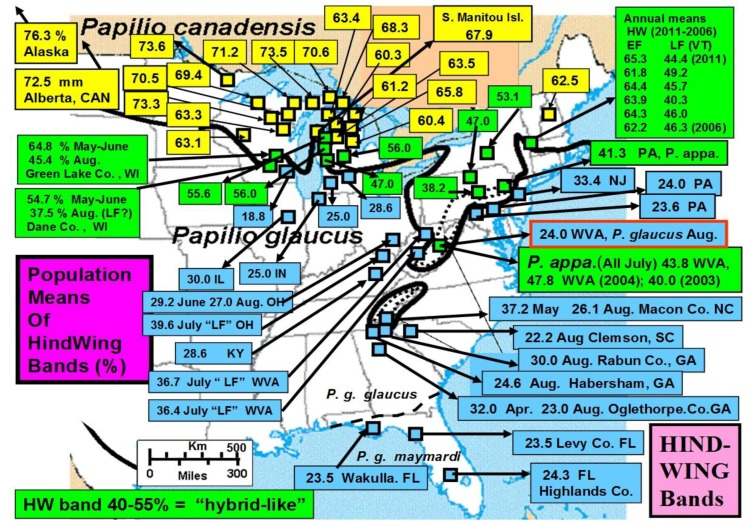
The mean annual male hindwing band widths of selected *Papilio* populations of *P*.* glaucus* (blue, 10%–44%), *P*. *canadensis* (yellow, 40%–55%), and their hybrids (green, 40%–55%, showing the historical hybrid zone (dark line), which basically has been delineated by the 2,600 Degree-days on the thermal landscape (see Figure 2 and Figure 3). The putatively univoltine *P*. *appalachiensis* (the hybrid species) is indicated in West Virginia (Pendleton Co., West Virginia, USA) as is the second generation males of *P*. *glaucus* (found just 1,000 m lower in elevation below Spruce Knob, where more D-days accumulate annually) flying in August, 2–4 weeks later. Mean HW bands of the Vermont recombinant hybrids “LF” and the early flight at this site are indicated for the 2006–2011 years (see also Table 2).

**Figure 14:**
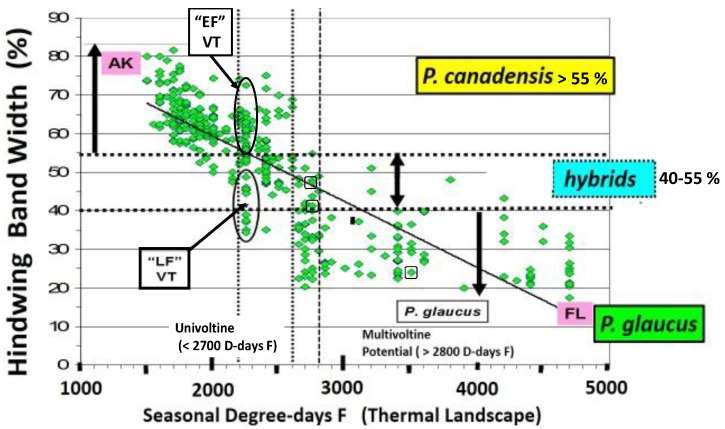
The mean annual male hindwing bands of *P*. *glaucus*, *P*.* canadensis*, and likely hybrids as a function of summer D-day (F) totals from Alaska (left, at 65° N latitude) to Florida (right; at 27° N latitude). The Vermont “LF” (late flight homoploid recombinant hybrids, in July) and the EF (early flight of *P*. *canadensis* in mid-May to June) are basically sympatric, but separated temporally by 3–5 weeks [24,100]. The hybrid species, *P*. *appalachiensis* is indicated by squares at 2,800 D-days, with the nearly sympatric *P*. *glaucus* of lower elevations at 3,500 D-days in Pendleton Co. The correlation is significant (R² = 0.6669, *n =* 277 population means).

**Figure 15:**
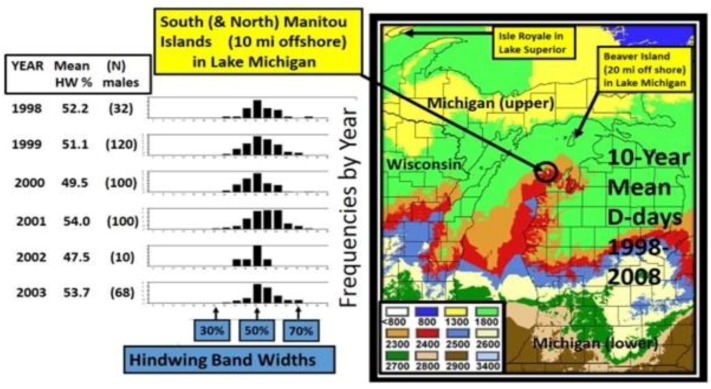
South Manitou Island in Lake Michigan appears to serve as a refuge for recombinant hybrid genotypes of *Papilio* despite being at high latitudes (45° N). Hindwing bandwidths of males are clearly “hybrid-like” (between 40% and 55%; see also Figure 13 and Figure 14), though they have increased slightly in 2006–2008 (57.7%, *n =* 131; 55.6%, *n =* 95; and 58.1%, *n =* 82, respectively). The 10-year average Degree-day accumulations across Wisconsin and Michigan reflect climate warming during 1998 to 2008, and the warmer western coast if Michigan. This island population also supported other hybrid-like traits, including *glaucus*-like tulip tree detoxification abilities, Z-linked allozymes (Pgd-100), and the enabler gene (b+) for female melanism [24,150,281,363] (see also Figure 16a,b). Such evidence of introgression was not found in other island populations on Beaver Island to the North, or the Isle Royale in Lake Superior ([462]).

**Figure 16:**
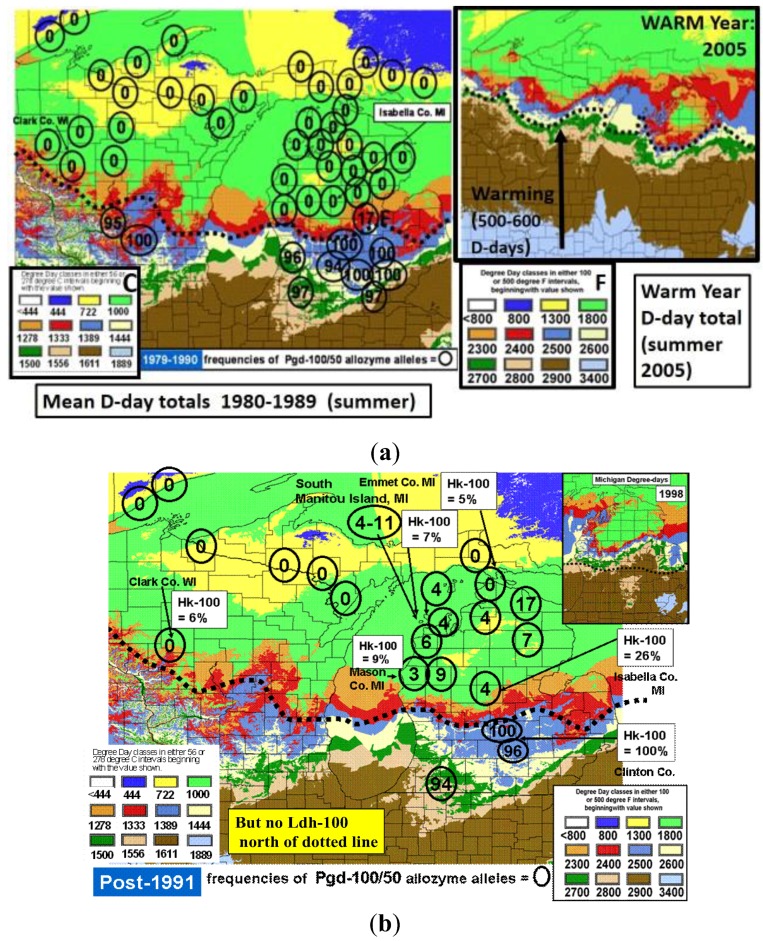
(**a**) Species diagnostic Z-linked frequencies of allozyme alleles (Pgd-100, or 50) in Michigan *Papilio* populations before 1991, shown with the 10-year average thermal landscape in degree-days (°F). A warmer year is shown in the upper right. During this period, the Z-linked Ldh allozyme frequencies were similar to the Pgd allozymes shown here [332,365]; (**b**) Species diagnostic Z-linked frequencies of allozyme alleles (Pgd-100, or 50) in Michigan *Papilio* populations post-1991, shown with the 10-year average thermal landscape in degree-days (°F). A warmer year is shown in the upper right. Also shown for comparison are the frequencies of the autosomal “glaucus-like allozyme alleles of HK-100 (hexokinase), which were also at zero frequency north of the historical hybrid zone (dotted line) before 1991 (also see [332]). During the years after 1991, no “glaucus-like” Ldh-100 or Ldh-50 alleles were found north of this line (similar to that reported for the Vermont “LF” recombinant hybrid population), reflecting strong divergent selection on the different parts of the Z-chromosomes of hybrid zone populations [24,101,340].

The hindwing band width serves as a useful diagnostic trait for *P*. *canadensis* (10%–40% width of anal cell), *P*. *glaucus* (55%–90%) and their hybrids (40%–55%; Figure 11, Figure 12, Figure 13 and Figure 14, [24,150,330]). Multiple hybrid zone transects and multiple-trait analyses will help us better understand the mosaic trait selection across the Z-chromosomes and genomes of these parental and hybrid *Papilio* [24,26,27]. However, the summer thermal landscape does provide an excellent predictor of the geographical distribution of hybrids and parental species in this group. This is especially true for some high latitude islands such as South Manitou Island in Lake Michigan (Figure 15) and also for the eastern mountains of North America, including Vermont (Figure 14; Table 2). Although not as evident as in the late flight hybrids of the Battenkill River area of Vermont, the Midwestern USA transects of the “glaucus-like” Z-linked species-diagnostic allozymes (Pgd-100/50, and Ldh-100) and the diagnostic autosomal allozyme HK (hexo-kinase) show differential introgression during the past 2 decades, reflecting strong divergent natural selection within the hybrid zone of Wisconsin and Michigan (cf. Figure 16a,b). The lack of movement for Ldh-100 suggests that its linkage with the direct development trait (od-) on the Z-chromosome, leads to mortality in all areas with insufficient D-days to support two generations. In contrast, survival is permitted by recombinant hybrid late-flight genotypes as well as *P*. *canadensis*, which have the obligate diapause (od+) trait [24,100].

**Table 2:** Mean hindwing band widths and forewing lengths of *Papilio* males collected in the Battenkill River area of western Vermont (Bennington County) and adjacent (Cambridge, New York, in Washington Co.).

Year/Date	n	Early Flight [May–June]	“EF”	n	Late Flight [Mid–July]	“LF”
		HindWing Band (%)	Forewing Length (mm)		HindWing Band (%)	Forewing Length (mm)
1984	52	72.0	44.9	na		
1999	23	56.9	46.0	1	30	50
2000	125	54.7	47.1	35	39.4	50.6
2001	0	x	x	51	37.6	49.3
2002	154	56.1	48.9	13	34.3	50.6
2003	29	55.9	44.9	14	38.9	51.0
2004	205	63.3	47.0	12	48.8	51.4
2005	252	63.6	45.8	0	X	X
2006	116	64.3	46.6	75	46.3	50.1
2007	111	65.8	47.7	44	46.0	48.3
2008						
May25–June 2	138	63.9	46.7			
June 26–June 29	67	67.0	46.7			
1–6 July				77	45.3	47.6
9–16 July				159	40.3	47.7
16–23 July				51	42.4	48.3
27–31 July				23	40.2	48.2
2009	142	64.4	47.0	36	45.7	47.3
2010	222	61.3	45.9	6	47.2	49.2
2011	241	65.3	46.1	179	44.4	49.5

The *P*.* glaucus* diagnostic lactate dehydrogenase allozyme Ldh-100 is often linked closely with the non-obligate-diapause gene (od-) on the Z (=X) chromosome, while the genes for obligate diapause (od+) and Pgd-0125 or Pgd-80 are shared by *canadensis-*like and *appalachiensis*-like siblings [24,482]. Inside the hybrid zone (with thermal Degree-days constraints on development), all recombinant Z-chromosome traits occurring with od- would be eliminated immediately that year since none of the second generation would reach the pupal stage before leaves abscised and winter arrived [24,328]. This strong climate-driven ecological selection [100] inside the hybrid zone leaves only those univoltine recombinant hybrid backcross-like offspring that are linked with the *canadensis*-type obligate diapause (od+) gene. As shown in the hybrid populations at the Battenkill River area in Vermont, the hybrid species, *P*. *appalachiensis* is also presumed to be comprised of univoltine (late flight recombinants) with hybrid like traits (e.g., Hindwing bands at 40%–55%; Figure 12, Figure 13 and Figure 14). Of these univoltine recombinant hybrids with od+, some have a delayed post-diapause development and respond by having adult emergence from diapausing pupae delayed by 3–5 weeks. This effectively and immediately isolates them from both parental species that emerge 3–5 weeks earlier at those sites, or nearby [101]. Such late-emerging genotypes may be the result of a Z-chromosome factor causing a delay in the developmental transition from the phase of “diapause maintenance” to “diapause termination”, as recently shown for the European corn borer moths [483].

We are in the process of determining the sequences on the Z-chromosomes to determine if independent evolutionary origins of Late-Flight genotypes and hybrid species at different transects of the extensive *Papilio* hybrid zone might be occurring, as seen in *Lycaeides* butterflies [69]. Also, while extinctions of the univoltine *P*. *appalachiensis* and recombinant hybrid late flight incipient species may occur on the warm side of the hybrid zone, as they are “driven off” the mountain top refuges (in the southern Appalachian Mountains in GA, NC, and SC) or outcompeted (or genetically swamped) by ascending populations of the multivoltine *P*.* glaucus* with climate warming [24], we still have potential on the cooler side for continuing origins of these recombinant hybrid genotypes with post-diapause emergence delays along multiple hybrid zone transects. If so, then natural local extinctions of southern refugial mountaintop populations may not be a total loss from a North American continental perspective of this butterfly hybrid species diversity. 

Morphospecies evolution, introgressive recombinant hybridization and reproductive isolation in these *Papilio* can apparently arise within only a few generations in nature under severe thermal constraints and the associated strong divergent natural selection as seen across the hybrid zone [24]. At what stage of divergence such “incipient species” actually become species (or hybrid species) remains an important concept for evolueionbary ecology as well as conservation. The elegant long-term work on European corn borer moths (*Ostrinia nubilalis*) also suggests a significant role in evolutionary divergence due to a Z-linked post-diapause development delay in hybrids [276,277,483]. This Z-linked post-diapause delay gene may turn out to be more widespread in Lepidoptera and it could in fact represent a type of recombinant hybrid “speciation gene” for rapid temporal reproductive isolation.

## 23. Conclusions

In the future, insect conservation and diversity must actively address genetic aspects of biodiversity (cryptic biodiversity), including genomics of individuals, population genetics, cryptic species, community interactions with genetically key species throughout various ecosystems, and “landscape genetics”. Phylogenetic histories will also be increasingly important for understanding the context of taxonomic classifications regarding the capacity for ecological resilience and evolutionary flexibility across future landscape [484]. The dynamic balance between creation (and extinction) of “species” may not be as temporally asynchronous as previously thought, because “speciation with gene flow” has emerged more commonly than ever previously thought possible. Hybrid speciation in polyploid plants and homoploid (recombinant) hybrid speciation in animals can be quite rapid, as seen here with several Lepidoptera groups. Furthermore, hybrid introgression may enhance adaptation rates for translocations and genetic rescues, and may provide a logical way to speed up needed management responses to rapid environmental changes. Genetic novelty and hybrid speciation in insects can be catalyzed in natural hybrid zones by climate changes, as is shown here. Management challenges involving genetic diversity may become increasingly feasible as we continue to develop new genomic assessment tools [485,486]. Integration of genetics across all levels from individual genomes to ecosystem and landscape genetics will increasingly characterize the future management of biodiversity and cryptic biodiversity in general.
